# A Concrete Example of the One Health Approach in the Brazilian Unified Health System

**DOI:** 10.3389/fpubh.2021.618234

**Published:** 2021-06-04

**Authors:** Paulo César A. Souza, Maria Cristina Schneider, Margarida Simões, Ana Glória Fonseca, Manuela Vilhena

**Affiliations:** ^1^Department of Epidemiology and Public Health, Veterinary Institute, Federal Rural University of Rio de Janeiro, Rio de Janeiro, Brazil; ^2^Department of International Health, School of Nursing and Health Studies, Georgetown University, Washington, DC, United States; ^3^Institute of Collective Health Studies, Federal University of Rio de Janeiro, Rio de Janeiro, Brazil; ^4^Mediterranean Institute for Agriculture, Environment and Development (MED), University of Évora, Évora, Portugal; ^5^Department of Veterinary Medicine, Sciences and Technology School, University of Évora, Évora, Portugal; ^6^Department of Public Health, NOVA Medical School, NOVA University of Lisbon, Lisbon, Portugal

**Keywords:** One Health, health systems, primary care, transdisciplinarity, surveillance, Latin America

## Introduction

The vision that we are all connected in this world is not new. However, to respond to the current challenges the world is facing, an integrated vision where humans, animals, and environment are linked has never been so important (1). Coronavirus disease 2019 (COVID-19) is the most recent example of the complex threats of emerging infectious diseases. El Zowalaty and Järhult (2) discuss the COVID-19 outbreak in a *One Health* context, highlighting the need for the implementation of this approach to improve human health and reduce the emergence of pandemic viruses.

The definition of *One Health* as an “effort to collaborate across multiple disciplines on the local, national, and global level to achieve optimal health for people, animals, and the environment” (3) implies that multidisciplinary approaches in research, education, varied services, and policies could support evidence-based decision-making in health and help build different solutions for challenges in the animal–human–ecosystem interface.

For some countries, the collaboration among disciplines and sectors is already a reality as is the case of Brazil. From the 1970's with the National Rabies Program, created by an agreement between the Ministry of Health and the Ministry of Agriculture, data from both sectors started to be shared, and cases in humans, dogs, other domestic animals, and wild animals were treated in a joint effort (1). In the 1980's, some experiences of multidisciplinary residences in Primary Health Care were created just after Alma-Ata enabled the participation of different professionals to work side by side in special projects in low-income areas (4). However, it was in the 1990's, with the creation of the Brazilian Unified Health System (Sistema Único de Saúde—acronyms SUS in Portuguese), based on a strong emphasis on Primary Health Care, that the opportunity for diverse professionals, namely, veterinary doctors, to become part of a multidisciplinary team came through, mostly at the local level. Altogether, SUS can be considered as a good and practical example of the *One Health* approach in Latin America.

## Subsections Relevant for the Subject

The knowledge and performance of Veterinary Medicine as part of the health professions in Brazil, namely, in the *One Health* approach, is an experience to be shared. The SUS (5) is the practical evidence of a State policy, which operates in all areas of health, from primary care to high complexity healthcare services. Among the policies of the SUS in Primary Care, the Family Health Strategy (FHS) is a focal point throughout the entire Brazilian territory, with local coordination in the municipalities; and whose basic team is formed by a family medical doctor, a nurse, a nursing assistant, and community health agents working in a given territory (6).

In 2008, extended multidisciplinary teams were created involving more professions to support the Health Centers (in Portuguese “Núcleos de Apoio às Equipes da Estratégia da Saúde da Família”—NASF) (7). These teams formed by several health professionals, such as psychologists, occupational therapists, and veterinary doctors, support a certain number of NASF. The selection of the professionals within the NASF is done according to the epidemiological reality of the territory to be worked by each health team.

In Brazil, the veterinarian is recognized as a health professional by both the Ministry of Health and the Ministry of Education since 1993 and has been part of the NASF Teams since 2011 (8), working in close collaboration with physicians and other health professionals, bringing to the operation the concept and practicality of *One Health* (1). These multidisciplinary teams working at the local level are part of the objectives of Health Surveillance and its components of Epidemiological, Sanitary and Environmental Surveillance (9). Hence, each NASF team has different joint activities, such as identification of potential zoonosis emergency, joint outbreak investigation, discussion of specific zoonotic cases (food-related or animal/vector-borne), home visits to follow-up events in the animal–human interface, identification and control of vectors and pests in the area and inside homes, analysis of environmental changes caused by man and natural disasters, and defining prevention and control strategies. The NASF teams also collaborate in the preparation of health education and communication strategies for the local communities, through team discussions, performing interdisciplinary actions, and developing shared responsibility (10).

In regard to the COVID-19 pandemic (caused by the infection of severe acute respiratory syndrome coronavirus 2, SARS-CoV-2), veterinarians have been strongly involved throughout the country at the municipal level, mainly in health surveillance, as well as in health education, mostly in both food safety and food production guidance. Besides, veterinarians were considered as part of the program on strategic action “Brazil Counts With Me—Health Professionals,” focused on the training and registration of health professionals for the detection and epidemiosurveillance of COVID-19 patients (11).

Altogether, the recognition as a health professional and the work developed by veterinarians in the SUS, in Brazil reveal that this experience can serve as an example to other countries in the Latin Americas and in other parts of the world. Furthermore, Queenan et al. (12) reveals unique experiences and advantages of integrated human and animal health services, namely, National Services of Italy, Canada, and Kenya. All these examples feature collaborative efforts and strategies for others to pursue.

## Discussion

For several years, the United Nations and the European Union, through different mechanisms, have been driving various initiatives to implement the *One Health* approach (13).

This awareness prompted a bibliographic review uncovering more than 250 articles related to *One Health* worldwide, many of them related to the concept and the history; however, publications referring to the application of the concept increased in number after 2013 (1). The turning point for the application of the *One Health* concept was the preparation for the potential avian influenza H5N1 pandemic around 2005 and 2006, when several official documents addressing the importance of intersectoral collaboration and joint preparedness plans were made in the Americas, Southeast Asia, and other parts of the world (1). Yet again, another influenza pandemic occurred in 2009 (H1N1 viral isolate) for which the previous joint preparedness plans greatly contributed to manage the disease dissemination. Together, these plans and evolving versions enabled prompt and adequate responses to other zoonotic threats.

The first declaration of a Public Health Emergency of International Concern (PHEIC), since a new version of the International Health Regulations (IHR) was in place (2005), was pronounced during the H1N1 pandemic when a stronger global cooperation for risk assessment and capacity-building was deemed crucial. Alongside, advocating for a *One Health* approach to better prevent, detect, and respond to any pandemic potential threat (14).

The coronaviruses (CoV) with animal origin represent a continuous pandemic threat to global health security, as previous coronavirus crises can be traced back to 2003 with the emergence of SARS-CoV and also in 2012 when the Middle East Respiratory Syndrome coronavirus (MERS-CoV) created a novel challenge (2). Although concrete evidence is not available, other hypothesis based on the mutation rate of a specific viral gene and molecular clock analysis considers that interspecies CoV infection, crossing from bovine to humans, may have occurred in the late 1890's (15). This possible CoV outbreak caused a worldwide disruption and maintains the infamous designation of the Great Russian Flu pandemic.

With respect to the human–animal health interconnectedness, the World Health Organization defines a zoonosis as any infection naturally transmissible from vertebrate animals to humans (16) and has already included the COVID-19 pandemic, caused by SARS-CoV-2, in these zoonotic diseases class. However, and according to Haider et al. (17), no animal reservoir has yet been identified, considering this classification as premature. Haider and collaborators propose that COVID-19 should instead be classified an “emerging infectious disease (EID) of probable animal origin,” without compromising the importance of zoonoses and communicable diseases common to humans and animals as potential PHEIC that is well-known for more than 20 years. Forasmuch as the importance of zoonoses, Taylor et al. (18) estimated that around 70% of infectious hazard threats to public health have an interface with animals, confirmed by other studies that have demonstrated the importance of animal/human health interface and suggested the need for more comprehensive research (19).

Remarkably, WHO included the *One Health* approach in evaluations of the country core capacities to implement the IHR (20). The joint evaluation exercise, coordinated by WHO, has been performed by countries and by peer reviewers in the context of the Joint External Evaluation (JEE), in order to evaluate the country core capacities to prevent, detect, and respond to possible PHEIC and include several indicators to evaluate this coordination (21). The most recent example of this assessment is related to COVID-19, a declared PHEIC by WHO, revealing each country/region's capacities and fragilities to address the pandemic and calling out for multisectoral collaborations. This could be considered one step further in the direction of the *One Health* approach and operationalization, setting the goal in truly understanding how animals and humans are linked, based on concrete examples. Noteworthy, it is not a current practice in most countries as demonstrated in the WHO/JEE scores (22).

Transdisciplinary studies and integrative collaboration across research, practice, and society counterparts are needed both to prompt a wholesome perspective (from local to global settings) and to enhance a comprehension of the details intertwined. The term “transdisciplinary health” was tentatively proposed by Assmuth et al. (23) to signify the multidimensional integration across fields relevant for health assurance. And what is the *One Health* approach really about? It is a framework that equates the shared environment affected by the socio-economic interest of humans. A *One Health* concept calls for various disciplines to work together to provide new methods and tools for research and implementation of effective services to support the formulation of norms, regulations, and policies to the benefit of current and future generations. This will improve the understanding of health and disease processes as well as the prediction, detection, prevention, and the control of infectious hazards and other issues affecting health and well-being in the human–animal–ecosystem interface, contributing to the sustainable development goals and to the improvement of equity in the world (1). Public Health depends on it!

## Author Contributions

All authors contributed for the article with opinions and discussion.

## Conflict of Interest

The authors declare that the research was conducted in the absence of any commercial or financial relationships that could be construed as a potential conflict of interest.
